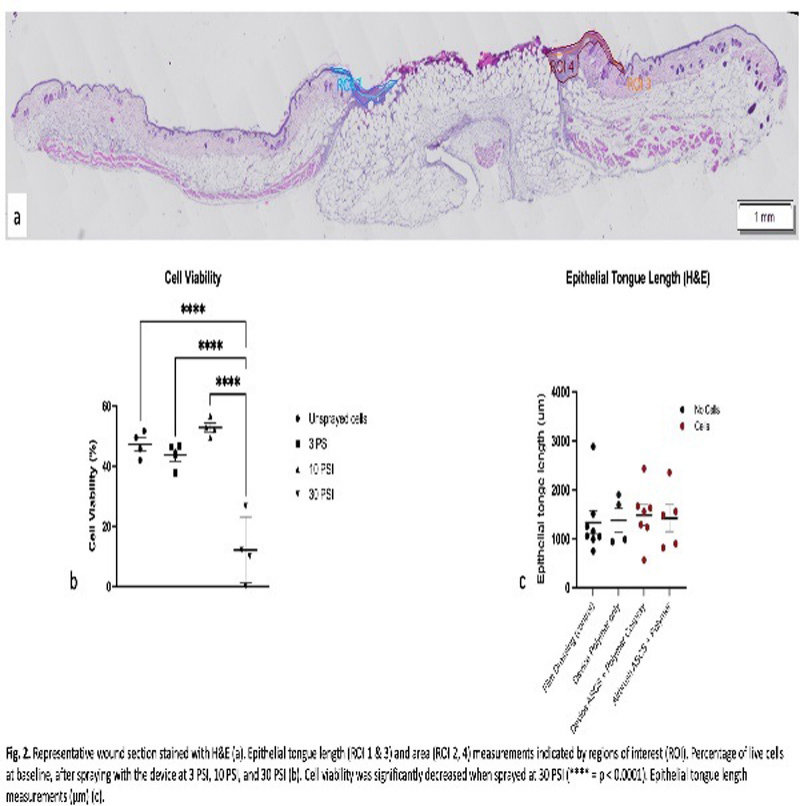# 71 A Novel Solution-Blow-Spinning Device Co-Sprays Autologous Skin Cell Suspensions and Polymer for Full Thickness Wounds

**DOI:** 10.1093/jbcr/irad045.045

**Published:** 2023-05-15

**Authors:** Michele Saruwatari, Bonnie Carney, Tyler Salvador, Metecan Erdi, Peter Kofinas, Jeffrey Shupp, Reza Monfaredi, Anthony Sandler

**Affiliations:** Children's National Hospital, Washington, District of Columbia; Firefighters' Burn and Surgical Research Laboratory, Medstar Health Research Institute, Georgetown University Medical Center, Washington, District of Columbia; Children's National Hospital, Washington, District of Columbia; University of Maryland, Bethesda, Maryland; University of Maryland, College Park, Maryland; Firefighters' Burn and Surgical Research Laboratory, MedStar Health Research Institute, Georgetown University School of Medicine, Washington, District of Columbia; Children's National Hospital and University of Maryland, Washington, District of Columbia; Children's National Hospital, George Washington University, Washington, District of Columbia; Children's National Hospital, Washington, District of Columbia; Firefighters' Burn and Surgical Research Laboratory, Medstar Health Research Institute, Georgetown University Medical Center, Washington, District of Columbia; Children's National Hospital, Washington, District of Columbia; University of Maryland, Bethesda, Maryland; University of Maryland, College Park, Maryland; Firefighters' Burn and Surgical Research Laboratory, MedStar Health Research Institute, Georgetown University School of Medicine, Washington, District of Columbia; Children's National Hospital and University of Maryland, Washington, District of Columbia; Children's National Hospital, George Washington University, Washington, District of Columbia; Children's National Hospital, Washington, District of Columbia; Firefighters' Burn and Surgical Research Laboratory, Medstar Health Research Institute, Georgetown University Medical Center, Washington, District of Columbia; Children's National Hospital, Washington, District of Columbia; University of Maryland, Bethesda, Maryland; University of Maryland, College Park, Maryland; Firefighters' Burn and Surgical Research Laboratory, MedStar Health Research Institute, Georgetown University School of Medicine, Washington, District of Columbia; Children's National Hospital and University of Maryland, Washington, District of Columbia; Children's National Hospital, George Washington University, Washington, District of Columbia; Children's National Hospital, Washington, District of Columbia; Firefighters' Burn and Surgical Research Laboratory, Medstar Health Research Institute, Georgetown University Medical Center, Washington, District of Columbia; Children's National Hospital, Washington, District of Columbia; University of Maryland, Bethesda, Maryland; University of Maryland, College Park, Maryland; Firefighters' Burn and Surgical Research Laboratory, MedStar Health Research Institute, Georgetown University School of Medicine, Washington, District of Columbia; Children's National Hospital and University of Maryland, Washington, District of Columbia; Children's National Hospital, George Washington University, Washington, District of Columbia; Children's National Hospital, Washington, District of Columbia; Firefighters' Burn and Surgical Research Laboratory, Medstar Health Research Institute, Georgetown University Medical Center, Washington, District of Columbia; Children's National Hospital, Washington, District of Columbia; University of Maryland, Bethesda, Maryland; University of Maryland, College Park, Maryland; Firefighters' Burn and Surgical Research Laboratory, MedStar Health Research Institute, Georgetown University School of Medicine, Washington, District of Columbia; Children's National Hospital and University of Maryland, Washington, District of Columbia; Children's National Hospital, George Washington University, Washington, District of Columbia; Children's National Hospital, Washington, District of Columbia; Firefighters' Burn and Surgical Research Laboratory, Medstar Health Research Institute, Georgetown University Medical Center, Washington, District of Columbia; Children's National Hospital, Washington, District of Columbia; University of Maryland, Bethesda, Maryland; University of Maryland, College Park, Maryland; Firefighters' Burn and Surgical Research Laboratory, MedStar Health Research Institute, Georgetown University School of Medicine, Washington, District of Columbia; Children's National Hospital and University of Maryland, Washington, District of Columbia; Children's National Hospital, George Washington University, Washington, District of Columbia; Children's National Hospital, Washington, District of Columbia; Firefighters' Burn and Surgical Research Laboratory, Medstar Health Research Institute, Georgetown University Medical Center, Washington, District of Columbia; Children's National Hospital, Washington, District of Columbia; University of Maryland, Bethesda, Maryland; University of Maryland, College Park, Maryland; Firefighters' Burn and Surgical Research Laboratory, MedStar Health Research Institute, Georgetown University School of Medicine, Washington, District of Columbia; Children's National Hospital and University of Maryland, Washington, District of Columbia; Children's National Hospital, George Washington University, Washington, District of Columbia; Children's National Hospital, Washington, District of Columbia; Firefighters' Burn and Surgical Research Laboratory, Medstar Health Research Institute, Georgetown University Medical Center, Washington, District of Columbia; Children's National Hospital, Washington, District of Columbia; University of Maryland, Bethesda, Maryland; University of Maryland, College Park, Maryland; Firefighters' Burn and Surgical Research Laboratory, MedStar Health Research Institute, Georgetown University School of Medicine, Washington, District of Columbia; Children's National Hospital and University of Maryland, Washington, District of Columbia; Children's National Hospital, George Washington University, Washington, District of Columbia

## Abstract

**Introduction:**

Autologous skin cell suspensions (ASCS) are useful for treating burn wounds, including in adjunct with split thickness skin grafts (STSG) for full thickness injuries.

Careful wound care is crucial after ASCS application to prevent leakage out of the wound bed. Solution blow spinning (SBS) can be used to spray polymer fiber mats with varied flexibility, adhesion, and absorption. Wounds treated with ASCS and SBS dressings demonstrate equivalent re-epithelialization as those treated conventionally. SBS polymers are currently applied using commercial airbrushes with limited control of sterility, pressure, and deposition rate, and have only been evaluated when applied after ASCS. This work investigates a novel SBS prototype designed to address these limitations and simultaneously spray cells and dressings.

**Methods:**

A SBS prototype was engineered combining a 3D printed, biocompatible, disposable dual-chambered nozzle, a reusable handheld module with programmable motorized actuators, and a pressure gauge connected to an external air source.

The polymers poly(lactide-co-caprolactone) (PLCL), which exhibits excellent adhesion in moist environments, and poly(lactic-co-glycolic) acid (PLGA), which conforms to irregular surfaces as a protective layer, were chosen for initial testing. Six mm full-thickness punch biopsies were created on the dorsal aspect of C57/BL/6 mice and stented (**Fig 1b**). ASCS was prepared from donor mouse skin and viability was tested before and after spraying at 3, 10, and 30 PSI. Wounds were dressed with either conventional transparent film adhesive dressing (**Fig 1c**), polymers sprayed using the device, ASCS+polymers sprayed using the device (**1e**), or ASCS+polymers sprayed using an airbrush (**1f**). Wounds were harvested on postoperative day 5, formalin fixed and paraffin embedded. Six µm sections were stained with hematoxylin and eosin (H&E), imaged (**Fig 2a**). Epithelial tongue length was measured by a blinded assessor.

**Results:**

ASCS cell viability was 47.3±8.3% at baseline and was only significantly decreased after spraying at 30 PSI (12.2±11.0%). Hence, 10 PSI was used for the mouse experiment (**Fig 2b**). Overall, there were no significant differences in epithelial tongue length between the wounds treated with different cell and polymer-based dressings (**Fig 2c**).

**Conclusions:**

The SBS prototype can be used to spray ASCS at pressures up to 10 PSI without significant loss of viability. The novel device was able to co-spray ASCS and polymers onto a wound bed. Wounds dressed with SBS polymers demonstrate equivalent epithelial tongue length at post-wound day 5 compared with wounds treated with transparent film dressing and airbrush controls.

**Applicability of Research to Practice:**

Currently, ASCS and dressing application are performed in a stepwise fashion, which allows the ASCS to leak out of the wound bed. A simultaneous application process could limit ASCS loss, streamline the wound care procedure and improve healing outcomes.